# Do Freshwater and Marine Bivalves Differ in Their Response to Wildfire Ash? Effects on the Antioxidant Defense System and Metal Body Burden

**DOI:** 10.3390/ijerph20021326

**Published:** 2023-01-11

**Authors:** Fátima Jesus, Filipa Mesquita, Elisa Virumbrales Aldama, Ana Marques, Ana M. M. Gonçalves, Luísa Magalhães, António J. A. Nogueira, Ana Ré, Isabel Campos, Joana Luísa Pereira, Fernando J. M. Gonçalves, Nelson Abrantes, Dalila Serpa

**Affiliations:** 1 CESAM—Centre for Environmental and Marine Studies, Department of Environment and Planning, University of Aveiro, 3810-193 Aveiro, Portugal; 2 CESAM—Centre for Environmental and Marine Studies, Department of Biology, University of Aveiro, 3810-193 Aveiro, Portugal; 3 Faculty of Veterinary and Experimental Sciences, Catholic University of Valencia, Calle Guillem de Castro 94, 46001 Valencia, Spain; 4 University of Coimbra, MARE-Marine and Environmental Sciences Centre/ARNET—Aquatic Research Network, Department of Life Sciences, 3000-456 Coimbra, Portugal

**Keywords:** oxidative stress, wildfire ash, enzymes activity, metal body burden, filter-feeders

## Abstract

Wildfires constitute a source of contamination to both freshwater and marine ecosystems. This study aimed to compare the antioxidant defense response of the freshwater clam *Corbicula fluminea* and the marine cockle (*Cerastoderma edule*) to wildfire ash exposure and the concomitant metal body burden. Organisms were exposed to different concentrations (0%, 12.5%, 25%, 50%, and 100%) of aqueous extracts of Eucalypt ash (AEAs) from a moderate-to-high severity wildfire. The activity of various enzymes, as well as lipid peroxidation, protein content, and metal body burden, were determined after 96 h of exposure. A significant increase in the protein content of soft tissues was observed for *C. edule* at AEA concentrations ≥ 25%, unlike for *C. fluminea*. Similarly, significant effects on lipid peroxidation were observed for cockles, but not for clams. For both species, a significant effect in the total glutathione peroxidase activity was observed at AEA concentrations ≥ 25%. Relative to the control, AEAs-exposed clams showed higher Cd content, whereas AEAs-exposed cockles showed higher Cu content, thus exhibiting different responses to the exposure to wildfire ash. The susceptibility of bivalves to ashes, at environmentally relevant concentrations, raises concern about the effects of post-fire runoff to bivalve species.

## 1. Introduction

Wildfires constitute a source of diffuse contamination to aquatic systems, mainly because post-fire runoff typically transports hazardous substances, like metals and polycyclic aromatic hydrocarbons (PAHs), associated with ash and soil particles [1,2]. As a consequence, wildfires have been reported to affect aquatic communities [2], for instance by decreasing the density, richness, feeding, and reproduction of macroinvertebrates [3,4,5], reducing fish abundance [4], and affecting the growth of primary producers [6,7]. Bivalve species are also among the aquatic biota susceptible to be affected by fire-related contaminants. Indeed, their reduced mobility, allied to their filter- and pedal-feeding behavior and benthic life cycle, might contribute to increased susceptibility to ash-associated contaminants in post-fire runoff [8].

However, the effects of post-fire runoff on this group of organisms have been largely overlooked [2]. From the few existing studies, Brito et al. [9] observed no acute toxicity on the mollusk *Biomphalaria glabrata* exposed to ash extracts. Specimens of the clam *Corbicula fluminea* exposed to ash extracts, on the other hand, exhibited acute toxicity (mortality) and accumulation of ash-associated contaminants in the soft tissues and, less extensively, in the shells [10]. In an in-situ study with *C. fluminea*, the feeding activity of the individuals was unaffected by post-fire runoff [11]. It is worth mentioning that these previous studies have all evaluated the effects of fire-related contaminants on freshwater bivalve species, whereas no studies have ever addressed the impacts on marine bivalves. Although wildfires typically ravage forested catchments, they can also affect coastal water bodies, as reported after the 2008 Central California (Big Sur) wildfires [12] or the recurrent wildfires in Galicia (Spain), which had devastating impacts on the fishing and aquaculture industries [13]. Ash transported to the water bodies can deteriorate the water quality by increasing organic matter and nutrient loads, turbidity, and the concentration of toxic compounds (e.g., [2,4,7]). As a consequence, forest fires were reported to cause significant losses in marine wildlife, namely bivalves, with negative impacts to the shellfish industry [13]. Freshwater aquaculture is also expected to be negatively affected by wildfires since a decrease in fish abundance has been reported after post-fire runoff (e.g., [2,4]).

At the sub-individual level, wildfire runoff was already proven to significantly affect biochemical responses of the aquatic biota. Exposure to contaminants is commonly linked to changes in the activity of antioxidant enzymes, which protect cells against oxidative damage by maintaining reactive oxygen species (ROS) at non-damaging levels (e.g., [14]). Exposure to wildfire ash or post-fire runoff affected the activity of several enzymes of the antioxidant defense system (e.g., catalase, glutathione peroxidase, glutathione S-transferase) and cholinesterases on freshwater invertebrates [15,16] and fish [17,18]. Such effects suggest oxidative and neuronal stress of ash-exposed organisms.

In this study, we aimed to compare the responses of a freshwater (*C. fluminea*) and a marine bivalve (*Cerastoderma edule*) to ash exposure at environmentally relevant concentrations. Different concentrations of aqueous extracts of ash were considered, aiming to mimic the dilution effect during runoff events [19]. The study focused on biochemical responses (antioxidant defense system), oxidative membrane damage (lipid peroxidation), protein content, and metal body burden. The response of the following oxidative stress enzymes was considered: catalase (CAT), total and selenium-dependent glutathione peroxidase (tGPx and Se-GPX, respectively), glutathione reductase (GR), and glutathione-S-transferase (GST). Lipid peroxidation (LPO) was also evaluated through determination of thiobarbituric acid reactive substances (TBARS). Metal content in soft tissues was measured to assess whether ash-associated contaminants accumulated in soft tissues and to establish direct links between the effects noticed and the dose of particular elements reaching physiological targets. As wildfires are known for increasing sediment transport from terrestrial to aquatic ecosystems, infaunal filter feeders commonly found in inland and coastal waters were selected for this study, since these organisms have a high capacity to bioaccumulate chemical substances bound to suspended particles as those dissolved in water [20,21]. Both selected species, *C. fluminea* and *C. edule*, as all are filter feeders, have high ecological relevance due to their key role in aquatic food webs and ecosystem dynamics. *C. fluminea* is an invasive freshwater clam, native to Southeast Asia, that has spread worldwide [22]. It is found in lotic and lentic sandy mud habitats, both pristine and polluted [22,23], owing to its tolerance to pollution [22,23,24]. *C. edule*, commonly known as cockle, is frequently found on sand, sandy mud, and fine gravel bottoms along the northwest European coasts and estuaries [25,26]. As both species are consumed by humans [20,25], the results of the present study are relevant not only in terms of environmental but also human health. Indeed, the consumption of bivalves contaminated with metals (namely As, Cr, Cd, Pb, and Hg) has been associated with mutagenic, carcinogenic, and genotoxic effects on human health [27]. To prevent such a risk for the population, international organizations like EFSA and FSANZ established maximum levels (MLs) of metal concentrations, above which edible seafood, including bivalves, cannot be sold for human consumption [27].

To the best of our knowledge, this is the first study addressing the metal body burden and the effects on the antioxidant defense system of a marine bivalve species exposed to wildfire ash. This, allied with the expected increase in wildfire frequency and severity as a result of climate changes [28], as well as the increasing number of fire-affected coastal waterbodies [29], highlights the timely character and relevance of the present study.

## 2. Materials and Methods

### 2.1. Collection of Test Organisms

*C. fluminea* individuals were collected in May 2022 in a shallow river (Mira, Portugal: N 40°21.5073′; W 8°42.4283′) and immediately transported to the laboratory in local water. Clams were kept in 20 L containers and gradually acclimated to laboratorial conditions (dechlorinated tap water continuously aerated; 20 ± 1 °C; 16 h light: 8 h dark photoperiod) for at least 15 days before the experiments. Culture water was renewed twice a week, and the clams were fed ad libitum with a concentrated suspension of the microalgae *Raphidocelis subcapitata*.

*C. edule* individuals were collected in May 2022 at the Ria de Aveiro (Aveiro, Portugal: N 40°38.5206′; W 8°44.1284′ and immediately transported to the laboratory. Cockles were transferred to glass aquaria containing seawater (previously filtered and adjusted to salinity 20 PSU) and kept at 20 ± 1 °C under continuous and intense aeration under a 16 h light: 8 h dark photoperiod. Organisms were acclimated to these conditions for 2 days, in the absence of food. After this period, they were fed with a mixture of rotifers and marine microalgae (obtained from Ocean Nutrition^®^ as frozen pellets) ad libitum. The culture water was renewed every other day.

Immediately upon arrival to the laboratory, 10 organisms of each species were randomly selected for further determination of the condition indices, biochemical responses, and metal body burden.

### 2.2. Preparation of Aqueous Ash Extracts (AEAs)

Ashes used in the experiments were collected in a eucalypt plantation at the Albergaria-a-Velha municipality (Aveiro district, North-Central Portugal) that was burned by a moderate-to-high severity wildfire in early September 2019 [30]. Immediately after the wildfire became extinguished, ashes were sampled and transported to the laboratory under dark and refrigerated conditions. Then, they were sieved through a 2-mm mesh sieve before being homogenized and stored at −20 °C until the preparation of the AEAs following previously optimized protocols [19,31]. 

Prior to the preparation of the AEAs, ashes were dried at 30 °C for 24 h. The AEAs were prepared by mixing the ash with the respective culture medium for each bivalve species [19], i.e., dechlorinated tap water for *C. fluminea*, and seawater adjusted to 20 PSU for *C. edule*. A maximum ash concentration of 10 g L^−1^ was selected for the present work, as in previous works [19,31], since total suspended solids concentrations of 5–10 g L^−1^ were measured in runoff waters from different burnt areas in North-Central Portugal during the first post-fire rainfall events (unpublished data).

The suspension was prepared in glass Erlenmeyers, which were wrapped in aluminum foil for protection against light, thus avoiding potential photodegradation of ash-associated contaminants. The mixture was placed in an orbital shaker for 2 h at 200 rpm, and then allowed to deposit for 12 h [10]. The aqueous fraction was obtained by siphoning the supernatant, being used immediately.

### 2.3. Ecotoxicological Tests

The ecotoxicological tests were performed following [10], with each species being exposed to five AEA concentrations: 0 (control), 12.5, 25, 50, 75, and 100%. Each concentration was prepared by diluting the AEA in the respective test medium for each species.

Experiments were carried out in glass beakers (1 L capacity) containing 500 mL of the respective test medium, continuously aerated, at the same temperature and photoperiod conditions as described for the acclimation period. For *C. fluminea*, 10 organisms per beaker were used, with shell length ranging from 18–19 mm (mean size 18.4 mm; SD = 0.23, *n* = 150). Individuals were fed daily with *R. subcapitata* at a concentration of 8 × 10^4^ cells mL^−1^. Regarding experiments with *C. edule*, 4 organisms per beaker were used, with shell length ranging 22–27 mm (mean size 24.9 mm; SD = 0.93, *n* = 80). The organisms were fed daily with a commercial mixture of rotifers and microalgae (Ocean Nutrition^®^) at a concentration of 3 mL L^−1^. The difference in the number of organisms per vial between both species had in consideration the required mass for the subsequent biochemical analysis accounting to possible mortality during exposure and further exclusion of organisms, as well as the acceptable density of individuals in the test vials [32,33].

The experiments were run for 96 h, and the test media was renewed 48 h after the start of the experiments. Standard water quality parameters, namely pH, dissolved oxygen, electric conductivity, and salinity were monitored in each beaker at the beginning and end of the experiment, as well as before and after renewal of the test media (Aquaread 800, Aquaread Ltd., Kent, England). Additionally, in the same days, samples of the test media were collected from each beaker and stored at −20 °C for analysis of metals and PAHs.

After exposure, seven randomly harvested organisms per treatment were measured (shell length) and weighted (total weight) and then dissected on ice to separate the soft tissues from the shells. Soft tissues were weighted and frozen at −80 °C for further biochemical analyses. Four random organisms per treatment were assigned to metal quantification. These organisms were transferred to a beaker containing clean medium (i.e., culture medium without AEA) and the same amount of food as given in the ecotoxicological tests, and a 2 h depuration period was allowed. This procedure aimed to allow the organisms to eliminate from their gut any AEA particles that could be represented in the chemical quantification that had not reached the target response systems. At the end of the depuration period, the organisms were measured, weighted, and dissected on ice: the foot was removed, and the rest of the soft tissue was weighted and frozen at −80 °C for further assessment of metal burden. The foot was not considered for quantification of metal burden, as it commonly shows low levels of metal accumulation in freshwater and marine bivalves [14,34].

### 2.4. Biochemical Analyses

The effects on the antioxidant defense system were evaluated in the soft tissue through quantification of the activity of oxidative stress enzymes (CAT, tGPx, Se-GPx, GR, and GST) and the lipid peroxidation through TBARS quantification. Soft tissues were homogenized in chilled phosphate buffer (50 mM, pH 7.0 with 0.1% Triton X-100) and the homogenized was centrifuged at 15,000× *g* during 10 mins. Then, the supernatants were collected in eppendorfs and stored at −80 °C for subsequent biochemical analyses. 

All enzymes’ activity was determined in seven individuals per treatment and were expressed relative to the protein content of the corresponding sample as nanomoles of substrate hydrolyzed per minute and per mg of protein, unless otherwise stated.

#### 2.4.1. Catalase (CAT)

CAT activity was evaluated by spectrometry according to Claiborne [35], with the adaptation to microplates described by Marques, Piló [21]. Briefly, 0.195 mL of phosphate buffer (0.05 M, pH 7.0) with hydrogen peroxide (H2O2; 0.010 M) were added to 5 µL of supernatant. The decrease of absorbance due to degradation of H2O2 was measured in UV-transparent microplates (flat-bottom microplates, Greiner Bio-One GmbH, Frickenhausen, Germany), at 240 nm (molar extinction coefficient of 43.5 M^−1^ cm^−1^) at 10 sec intervals during 3 min. Determinations were carried out in triplicate, considering the difference of the absorbance values obtained at t = 1 min and t = 3 min.

#### 2.4.2. Glutathione S-Transferase (GST)

GST (EC 2.5.1.18) activity was evaluated by spectrometry, according to Habig, Pabst [36]. Briefly, 200 µL of reaction solution (prepared using phosphate buffer 0.1 M, pH 6.5) were added to 100 µL of sample. The increase of absorbance due to formation of the thioether (molecular extinction coefficient of 9.6 mM^−1^ cm^−1^) was measured (a single determination per organism) in microplates at 340 nm during 5 min.

#### 2.4.3. Glutathione Reductase (GR)

GR (EC 1.8.1.7) activity was assayed by spectrometry, according to Carlberg and Mannervik [37], using a phosphate buffer (200 mM, with EDTA 2 mM, pH 7.0). The decrease of absorbance due to NADPH oxidation was measured (a single determination per organism) in microplates at 340 nm (molecular extinction coefficient of 6.22 mM^−1^ cm^−1^) during 5 min. 

#### 2.4.4. Glutathione Peroxidase (GPx) 

GPx (EC 1.11.1.9) activity was determined by spectrometry according to Flohé and Günzler [38], using a phosphate buffer (100 mM, pH 7.0). This method is based on following the NADPH oxidation (molecular extinction coefficient of 6.22 mM^−1^ cm^−1^) while oxidized glutathione is reduced back to the reduced form (reduced glutathione) by glutathione reductase. Both total GPx (tGPx) and selenium-dependent GPx (Se-dependent GPx) activities were determined, using cumene hydroperoxide (0.7 mM) and hydrogen peroxide (0.255 mM) substrates, respectively. Both activities were measured in microplates at 340 nm (molecular extinction coefficient of 6.22 mM^−1^ cm^−1^) during 5 min. 

#### 2.4.5. Thiobarbituric Acid Reactive Substances (TBARS)

TBARS were determined by spectrometry according to Buege and Aust [39]. This method is based on the reaction of lipid peroxidation by-products (such as malondialdehyde, MDA) with 2-thiobarbituric acid. The amount of TBARS was evaluated at 535 nm (molar extinction coefficient of 1.56 × 10^5^ M^−1^ cm^−1^) as a single determination, and results were expressed as nmol of MDA equivalents per mg of sample protein.

#### 2.4.6. Total Protein Concentration

The total protein concentration of each sample was determined by spectrometry at 595 nm, according to the Bradford method [40], adapted to microplates, using γ-bovine globulin as a standard. Protein concentration was determined in quadruplicate.

### 2.5. Determination of the IBRv2

The stress index ‘‘integrated biomarker response version 2” (IBRv2) was determined using the results of the abovementioned biomarkers (Section 2.5) to provide a holistic overview of the stress response of each species. The IBRv2 was calculated for both species, according to Sanchez, Burgeot [41]. For each biomarker, individual data (Xi) were divided by the control mean value (X0), followed by a log transformation to reduce variance (Yi = log (Xi/X0). Then, data were standardized (Zi = (Yi − l)/r, being I the general mean, and r the standard deviation); finally, the biomarker deviation index (A) was calculated subtracting Z0 (Z value for the control treatment) to the Zi values (A = Zi − Z0). The A values, representing the deviation of each biomarker relative to the control, are presented in a star plot. The IBRv2 values for each biomarker at each AEA concentration were obtained by summing up the absolute A values (IBRv2 = sum|A|). 

### 2.6. Condition Index

The condition index (CI) is a widely used parameter in ecological studies with bivalves, being used as an indicator of the global physiological status of the organisms, with lower values commonly representing a poorer physiological status (e.g., [42]). Herein, CI was determined based on the soft tissue wet weight and shell length, as defined by Kagley and Snider [43]:

$$\mathrm{CI}=\frac{\mathrm{soft}\;\mathrm{tissue}\;\mathrm{wet}\;\mathrm{weight}\;\left(g\right)}{\mathrm{shell}\;\mathrm{length}\;\left(\mathrm{mm}\right)}\times 100$$

### 2.7. Chemical Analyses

Water samples (three replicates per treatment, both in freshly prepared and 48 h old media) were collected after homogenization and then stored at −20 °C until being analyzed for PAHs and metal concentrations. The 16 priority PAHs defined by the Environmental Protection Agency (USEPA): acenaphthene, acenaphthylene, anthracene, benzo(a)anthracene, benzo(a) pyrene, benzo(b)fluoranthene, benzo(k)fluoranthene, benzo(g,h,i)perylene, chrysene, dibenzo(a,h)anthracene, fluoranthene, fluorene, and indeno(1,2,3-cd)pyrene were determined by gas chromatography coupled to mass spectrometry (GC-MS), according to the DIN 38407-39 (F39). Water samples for metal quantification were acidified (concentrated HNO_3_, 65% Suprapur^®^ for trace analysis, Supelco^®^) and metal concentration was determined for V, Cr, Mn, Fe, Co, Ni, Cu, Zn, As, Cd, and Pb by inductively coupled plasma-mass spectroscopy (Thermo X series) according to ISO 17294. The obtained recoveries ranged between 90% and 110% for all samples. The quantification limits of PAHs and metals for both the freshwater and the brackish media are presented in Table S2 (Supplementary Material) and Table 1, respectively.

Biological samples (soft tissue excluding the foot, 4 replicates per treatment) were analyzed for metal concentrations. PAHs concentrations were not quantified as their concentrations in the water samples were consistently below quantification limits. Tissue samples were allowed to dry at 105 °C during 24 h and then weighted. The samples were transferred to Teflon tubes and digested overnight at 60 °C with 3 mL of concentrated HNO_3_ (65% Suprapur^®^ for trace analysis, Supelco^®^). Afterwards, 0.5 mL of hydrogen peroxide (Suprapur, 30%, Merck KGaA, Darmstadt, Germany) was added and the samples were kept at 60 °C during 2 h. This latter procedure was performed 3 times (1.5 mL of hydrogen peroxide added in total). The cooled samples were then analyzed by ICP-MS (Thermo X series) according to ISO 17294, as mentioned above.

All chemical analyses were performed by accredited external laboratories.

### 2.8. Dietary Hazard Assessment

Since both *C. edule* [25,26] and *C. fluminea* [20,44] are consumed as seafood, and taking into consideration the high propensity of bivalves to accumulate contaminants (e.g., [21,24]), the potential hazard associated with the consumption of contaminated individuals is addressed herein as a complement to the effects assessment. The metal concentration in the soft tissue of each bivalve species (wet weight) was compared to the maximum levels set by different organizations (EFSA—European Food Safe Authorities; FSANZ—Food Standards Australia and New Zealand). Furthermore, the amount (kg) of bivalves to be consumed per week to exceed PTWI (Provisional Tolerable Weekly Intake, fixed by the Joint FAO/WHO Expert Committee on Food Additives) was also defined, considering a 70 kg adult [45].

### 2.9. Data Treatment and Statistical Analyses

To test for significant differences between organisms exposed to different AEA concentrations, and those from the test controls, regarding the endpoints Condition Index, antioxidant enzymes activity, TBARs, protein and metal concentrations, data were analyzed through the parametric one-way analysis of variance (ANOVA), followed by the multiple comparisons Dunnett’s test. Data were previously checked for normality (Shapiro–Wilk test) and homoscedasticity (Brown–Forsythe test). If ANOVA assumptions were not met, the non-parametric Kruskal–Wallis, followed by Dunn’s test, was used instead. A level of significance of 0.05 was used for all statistical tests.

## 3. Results and Discussion

### 3.1. Chemical Analyses of the Exposure Media

The parameters pH, dissolved oxygen, conductivity, and salinity measured in the test vials are presented in Table S1. No relevant changes were noticeable regarding these parameters through the experiments. Concerning differences among treatments, only conductivity and salinity increased with the increase in AEA concentration, as expected.

PAHs concentrations in the water samples were below quantification limits. This is not surprising, as previous studies using Eucalypt AEAs from high severity wildfires reported only the presence of naphthalene [46,47] and phenanthrene [48], at concentrations < 0.3 µg L^−1^, but AEAs were prepared using higher ash concentrations.

Metal concentrations in the test media are presented in Table 1. As for the metals, As, Cd, and Pb were consistently below quantification limits, whereas Fe was only found in the freshwater media at the AEA concentration of 100% (new medium). In the brackish water samples, only Mn and Co were found, and only in the new media, most likely due to the higher quantification limits compared to freshwater.

In general, in the freshwater media, there was a trend for similar or decreased metal concentrations in the 48 h old media compared to freshly prepared media, except for Zn and, to a minor extent for Co, with the latter presenting higher concentrations in the 48 h old media. The trend for decreased metal concentration in the medium over a 3 d exposure to AEAs was also observed in a previous study with *C. fluminea* [10], which is likely related to metal uptake from the medium. Interestingly, these authors found increased concentrations of some metals in the 3 d old medium, compared to freshly prepared medium, namely for Ca, Mo, and Na, among others, but not for Zn. Remarkably, after a 7 d exposure period this trend was commonly inverted, i.e., compared to the freshly prepared medium, and the concentrations of these metals increased after 3 d of exposure but decreased after 7 d of exposure [10]. Thus, we cannot discard the possibility that both Zn and Co concentrations could possibly decrease after a longer exposure period. Regarding Zn, which showed a more pronounced concentration increase, the observed trend will likely be related to excretion, as Zn concentration in the soft tissue was found to decrease with increasing AAE concentration, most pronouncedly at 100% AEA (see results in Section 3.4).

The metal concentrations in AEAs reported in the present study are lower than those reported in a previous study with Eucalypt AEAs from a high severity wildfire [19], which might be related to a different origin of the ashes and to the variability promoted by the combustion conditions [46], vegetation type [19,47], among other factors. Still, there were similar trends, such as the metals showing higher concentrations, with Cu, Mn, and V among the most concentrated metals. Moreover, Cd was among the least concentrated metals in both the present study and the study by Santos, Abrantes [19]. Comparison to other studies using Eucalypt AEAs is hampered by the use of different ash concentrations [10,48,49].

### 3.2. Biochemical Analyses

**Figure 1:**
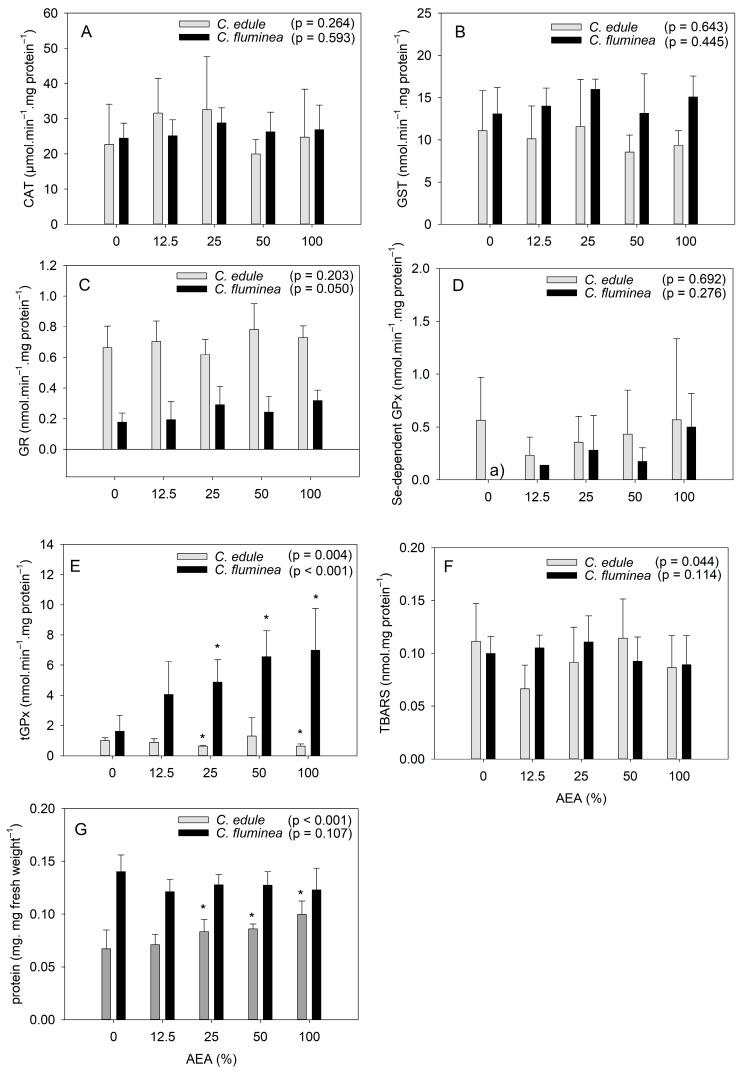
Biochemical responses of *Corbicula fluminea* and *Cerastoderma edule* individuals after 96 h of exposure to aqueous extracts of ash (AEA) at different concentrations (0, 12.5, 25, 50, and 100%): activity of the enzymes catalase (**A**), glutathione-S-transferase (**B**), glutathione reductase (**C**), total glutathione peroxidase (**D**), selenium-dependent glutathione peroxidase (**E**); production of thiobarbituric acid reactive substances (**F**); protein content (**G**). Bars represent mean values and error bars represent standard deviation. Asterisks stand for statistically significant differences relative to the control (0%) for each species. (a) The activity of this enzyme in control organisms was below the quantification limit of the method.

The oxidative stress response of both species was similar, differing only regarding the activity of tGPx, the amount of TBARS, and the protein content (Figure 1). CAT, GST, GR, and Se-dependent GPx activities were not significantly affected by AEA exposure for either species (Figure 1). Regarding tGPx activity (Figure 1E), it was affected in both species. Interestingly, the response of tGPx differed between the species, since increasing AEA concentration (≥25%) caused an increase in tGPx activity for the clams, but a decrease for the cockles. The amount of TBARS was significantly affected by the AEAs only for cockles (Figure 1F), suggesting potential lipid damage. However, the lack of significant differences relative to the control following post-hoc tests, as well as among treatments, indicates that these effects were only marginal (H(4) = 9.775, *p* = 0.044).

Regarding *C. fluminea*, the lack of response of both CAT and GST activity to AEAs exposure agrees with a previous study reporting that these enzymes did not allow for distinguishing between a contaminated site and a reference site [50]. The fact that no effect on the GR activity was observed agrees with a previous study reporting that this enzyme did not respond to contaminated sediments [51]. GPx, on the other hand, was reported to be susceptible to contamination, showing increased values in organisms inhabiting polluted sites [50], which agrees with the present results regarding tGPx activity. The fact that the activity of this enzyme was pronouncedly affected by exposure to the ash extracts suggests that the clams are more sensitive to ash-associated contaminants than the cockles. Still, considering that only tGPx activity was affected, allied to the lack of effects on TBARs content, suggests the occurrence of only minor effects under the tested laboratorial conditions. Such findings might be a result of the valve-closure behavior of clams when exposed to contaminants, as reported in previous studies (e.g., [11,52]), and concordantly to informal observations made during the laboratorial experiment. Indeed, clams exposed to AEAs at concentrations ≥ 50% were usually with their valves closed, unlike the control organisms and those exposed to AEAs at 12.5%. In opposition, cockles commonly had their valves open when exposed to any AEA concentration.

In what concerns to the response of *C. edule*, previous studies also reported that CAT activity [21,53], GST activity [21,33,53], and GR activity [21] were not responsive to contamination. Regarding TBARS, they have been reported to respond to increased Cu concentration [33] but not to an increased contamination level broadly [21]. Interestingly, in the study by Marques and Piló [21], only GPx activity varied significantly with the contamination level, which agrees with the significant effects found for tGPx activity in the present study. GPx is involved in the metabolism of reactive oxygen species (ROS), by reducing H2O2 to H2O (Se-dependent GPx) and organic peroxides to stable alcohols, using reduced glutathione as electron donor [12,54]. Commonly, GPx activity tends to increase with increased contamination (e.g., [21,33]), reflecting the need to maintain an elevated protection against the oxidative stress insult that environmental contaminants represent [54]. This agrees with the results of the present study regarding the clams, but not with the results observed for cockles. Indeed, the cockles showed inhibited activity of tGPx, which points out depressed defenses, denoting the failure of the organisms to counteract the oxidative toxicity of contaminants [54]. However, the herein observed activity inhibition was not pronounced (Figure 1E), which suggests a certain tolerance to ash-associated contaminants. In fact, the small, reduced activity of tGPx, allied to the slight trend for reduced TBARS content, suggests reduced oxidative stress of *C. edule* under the tested conditions. This is not surprising considering that *C. edule* has been considered a tolerant species to pollution compared to other marine bivalves [21,55], possibly due to adjustment mechanisms in the presence of contaminants. Still, given that tGPx activity was the enzyme showing the most pronounced response to ash exposure, we recommend its use in future studies as a possible biomarker of exposure to ash.

The present results suggest that cockles are less sensitive to ash-associated contaminants than the Asian clams, which agrees with the common trend of freshwater species to be more sensitive to metals than marine species [56], partially due to the variation of the chemical metal forms available in each media and their changes owing to the water physicochemical parameters (such as pH and salinity) [14], as well as competitive inhibition caused by hardness salts [57].

The fact that biochemical analyses were performed in the whole soft tissue, similarly to other studies (e.g., [21,53]), could have also influenced the results, since biochemical responses can be tissue-specific. As an example, the pattern of GST and GPx activities in *C. fluminea* showed differences when determined in gills compared to the digestive glands [52,58] or to the gonads [59]. Different response patterns between tissues can lead to a lack of statistical differences when whole-body homogenates are used [60]. For this reason, the present results should be interpreted with caution. 

**Figure 2:**
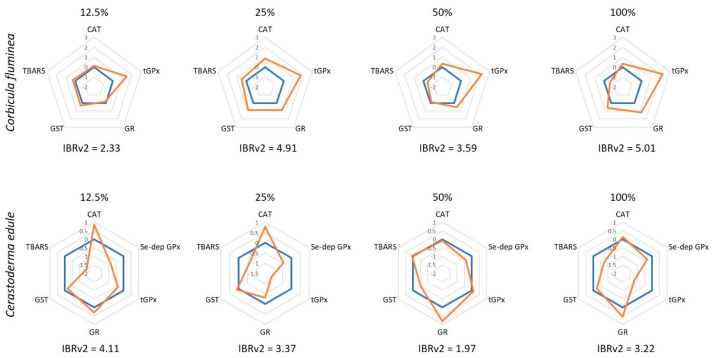
Integrated biomarker response (IBRv2) values of *Corbicula fluminea* and *Cerastoderma edule* individuals after 96 h of exposure to aqueous extracts of ash (AEA) at different concentrations (0, 12.5, 25, 50, and 100%). The orange line represents the IBRv2 index of AEAs-exposed organisms and is presented relatively to the control organisms (blue line; 0). Values above 0 indicate biomarker induction, whereas values below 0 indicate inhibition.

The IBRv2 results (Figure 2) showed that tGPx activity was pronouncedly induced in *C. fluminea* at all AEA concentrations. In opposition, changes were less pronounced in *C. edule*, with CAT being the most induced enzyme at low concentrations and GR at high concentrations. Based on the IBRv2 values, it is observed that *C. fluminea* was more sensitive than *C. edule* at all AEA concentrations, except at 12.5%, which agrees with the discussion above.

**Figure 3:**
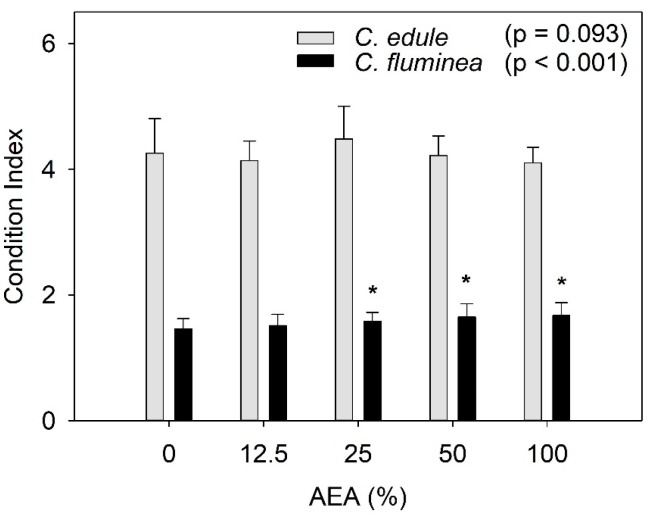
Condition index of *Corbicula fluminea* and *Cerastoderma edule* individuals after 96 h of exposure to aqueous extracts of ash (AEA) at different concentrations (0, 12.5, 25, 50, and 100%). Bars represent mean values and the error bars represent standard deviation. Asterisks stand for statistically significant differences relative to the control (0%) for each species.

The protein content of the cockles increased at AEA concentrations ≥25%, but it was unaffected in the clams (Figure 1G). These findings agree with a previous study that reported the protein content in *C. edule* to be positively correlated to metal concentrations in sediments, despite no statistically significant differences being found among sites with different pollution levels [53]. It is worth noting that *C. edule* showed higher protein content with increased AEA concentrations, but their Condition Index was not significantly affected (Figure 3). Given that their water content was not affected by the AEA concentration (Figure S1), this suggests that their content in lipids and carbohydrates might have changed to counteract the increased protein content, as hypothesized above. Taking into consideration that *C. edule* is an edible bivalve, with high value for the shellfish industry in different European regions, these findings suggest that exposure to ash might alter the nutritional value of this species.

It is also worth mentioning that under control conditions, the protein content of the clams was about twice that of the cockles (Figure 2G). The high nutritional value of clams might explain their consumption in several countries (e.g., [44]), despite their individual reduced mass relative to shell size.

### 3.3. Condition Index (CI)

The CI values of *C. fluminea* were significantly affected by the AEAs at concentrations ≥ 25%, showing a trend for increasing values with increasing AEA concentrations (Figure 3). In opposition, the CI values of *C. edule* were not significantly affected by exposure to the AEAs.

Since CI is a function of the fresh weight of the soft tissues, we hypothesized that the increased values might be due to a potential increase in the water content. To test this hypothesis, the water content of the soft tissues for both species was determined (Figure S1). Results showed that the increased CI values were not associated with an increase in the water content of the soft tissue. For both species, there was a slight trend for an increase in the tissues water content, but this was not statistically significant (*C. fluminea*: F_4, 20_ = 1.830, *p* = 0.163; *C. edule*: F_4, 20_ = 2.689, *p* = 0.061).

Increased CI values were also observed in *C. fluminea* after exposure to coal-fired power plant discharges compared to a reference site [61], in agreement with the present study, which denotes a better physiological status (e.g., [42]). Such an increase might be due to the availability of ash particles, which may act as a food source [61,62]. Indeed, ash contains both organic and inorganic particles [63], and since *C. fluminea* is known to feed on suspended particles [23], the improvement of their physiological status should not be unexpected. As the protein content of *C. fluminea* did not increase after exposure to the AEAs (Figure 1G), one might hypothesize that this increase in CI values might be related to an increase in the lipids and/or carbohydrates content, but this has to be tested in future research.

### 3.4. Metal Body Burden

Regarding metal internal concentration in the bivalves after the 96 h exposure period (expressed in a fresh weight basis), slight differences were observed between species. In *C. fluminea*, the most abundant metals were Fe and Zn, followed by Cu and Mn (Table 2). These results support the higher accumulation of Zn, Cu, and Mn by *C. fluminea*, as recently reviewed [24], and agrees with metal concentrations reported in previous studies (Table S3). In *C. edule*, the most abundant metals were also Fe and Zn, followed by Ni and As (Table 2), with the latter being not found in the clams. The high propensity of cockles to accumulate Zn, Ni, and As has also been reported in other studies ([21], cf. Table S3), [26,64]. In particular, *C. edule* was shown to accumulate higher concentrations of Ni compared to other marine species [21,65,66,67]. Note, however, that the internal metal concentrations in both species are not necessarily related to AEAs exposure, as explained below.

In *C. fluminea*, significantly lower Mn, Fe, Cu, and Zn concentrations in the soft tissues were found with increasing AEA concentrations, relative to the control organisms. This was not expected considering the trend for increased concentrations of Fe, Cu, and Mn with increasing AEA concentration (Table 1). However, the fact the concentration of these metals was higher at the beginning of the experiment than at the end of exposure suggests that clams were depurating or excreting during the experiment, despite an acclimation period of about 3 weeks which had been allowed. A previous study with *C. fluminea* also reported limited accumulation of some metals, namely Cu, in organisms exposed to metal-rich effluents [22]. Indeed, there is evidence that, when exposed to chemicals, *C. fluminea* has the ability to not only decrease uptake of contaminants from the outer medium but also to excrete them, which constitutes defense mechanisms. *C. fluminea* has been reported to decrease metal absorption when exposed to increased suspended solids concentrations, resulting in decreased accumulation [24]. Clams exposed to higher AEAs concentrations faced higher suspended solids concentrations, as deduced from the visual inspection of the exposure media (Figure S2). Moreover, as previously mentioned, *C. fluminea* is reported to close the valves when exposed to hazardous chemicals, which might also contribute to explaining reduced metal uptake at higher AEA concentrations. Furthermore, *C. fluminea* individuals, as other bivalve species, have the ability to use their mucous to depurate metals through sequestration and elimination as pseudofaeces [22] as well as to selectively reject particles, which represents a strategy to avoid internal exposure to toxic chemicals [22,32]. Rejected particles are involved in mucous, produced on the demibranchs, and expelled by the exhalant siphon without ingestion [22,32]. This is consistent with our observation that clams exposed to higher AEAs concentrations (50 and 100%) produced significant amounts of mucous, unlike those exposed to lower AEAs concentrations. Mucous was not addressed for metal quantification, but it may contribute to explaining the decreased metal body burden observed upon exposure to AEAs.

Considering the metal concentrations expressed as dry weight (Table S3), significant differences were found for Co (despite no significant differences relative to control), Cu, and Cd. Regarding Cd, data suggest accumulation, as the concentrations observed after the exposure to AEAs at 25% and 50% are higher than values in control organisms, as well as above values of the organisms representing the field condition (Table S3). It is worth noting that the clams accumulated Cd despite its low concentrations in the water (below 0.1 µg L^−1^, Table 1). The high accumulation of Cd in *C. fluminea* has been reported in previous studies ([70] cf. Table S3), [71]. The lower Cd concentrations in clams exposed to 100% AEA are likely related to the valve-closure behavior. It is also worth noting that Cd and Pb were only found in organisms exposed to AEAs (Table 2).

In the cockles, the concentrations of Cr, Mn, and Fe tended to increase with increasing AEA concentrations, although not in a significant manner. In particular, Cr was only found in cockles exposed to AEA concentrations ≥ 25% (Table 2). Considering the metal concentrations expressed as dry weight (Table S3), significant differences were found for Cu, with higher concentrations observed after exposure to AEAs at 50% and 100%, which suggests accumulation.

Comparing both species, the metal body burden of Cu, Mn, Fe, Co, Zn, Cd, and Pb (dry weight) was statistically higher in the clams’ soft tissues than in the cockles (Tables S3 and S5). The difference was particularly pronounced for Cu, as the concentration in the organisms exposed to 100% AEA was 12-fold higher in clams than in cockles. Conversely, the concentration of Co was significantly higher in the cockles. Moreover, Cr, Ni, and As were only found in the cockles, which agrees with previous studies reporting the ability of *C. edule* to accumulate higher concentrations of Cr and Ni compared to other bivalves [66,67].

In summary, the present results show that exposure to ash can affect the internal metal concentrations in the selected bivalves, as given by different metal concentrations in AEAs-exposed organisms compared to control organisms. However, it must be highlighted that internal metal concentrations after ash exposure were mainly determined by their concentrations previous to the experiment (field condition). This suggests that the assessment of the effects of exposure to wildfire ash on the metal body burden of bivalves must necessarily take into consideration the environmental metal concentrations in their habitats.

### 3.5. Dietary Hazard Assessment

Compared to the guidelines for human consumption, no metal raises concern for the clams. However, two metals, As and Ni, raised concern regarding a potential dietary risk for the cockles. The concentration of As in the cockles was 1.6 to 1.8-fold higher than the FSANZ guideline value (1 mg kg fresh weight^−1^, cf. Table 2). A previous study also showed that the only metal/metalloid with concentrations above the FSANZ guideline value in the *C. edule* collected at Ria de Aveiro was As [26]. Another study showed that As concentrations in *C. edule* were 20–33 µg g^−1^ dw, slightly above those found in the present study (15–15 µg g^−1^ dw, Table S3). Considering the PTWI (provisional tolerable week intake) of 0.015 mg kg^−1^ week^−1^ [45], the quantity of cockles (fresh weight of soft tissue) that a 70 kg adult needs to consume to exceed PTWI is 0.61 kg (Table S6). Considering the cockles immediately after collection, the value is 0.69 kg. Thus, although the dietary risk posed by cockles concerning As concentration is not related to AEAs exposure, it should be noted that exposure to ash might contribute to risks such as ash, which represent a source of As to the environment (Table 1).

Another metal that raises concern for human consumption is Ni, for which a consumption of 0.93 kg of cockles exposed to 100% AEA for 96 h (fresh weight of soft tissue) was needed to exceed the PTWI (Table S6). The strong ability of *C. edule* to accumulate Ni [26,64,66,67], as mentioned above, means that these organisms, when exposed to Ni, even at low concentrations, will accumulate this metal, with potential effects in human health. Note that Ni concentration in the water at 100% AEA was not high. Indeed, although Ni was not found in the cockles’ test media likely due to the high quantification limits (QL = 50 µg L^−1^, Table 1), it was found in ash and its concentration was below 3 µg L^−1^ in the freshwater (Table 1). Considering that ash used in both experiments was from the same batch, no pronounced differences in contaminant concentrations were expected.

Given the dietary risk posed by cockles, it is recommended that cockles used for human consumption should be regularly tested for their metal content, not only after specific pollution events, such as ash-loaded runoff events, but also in a routine basis to disclose any dietary risk. This recommendation is applicable to other bivalves.

Overall, the present study highlights that wildfire ash can negatively affect bivalve species, both from freshwater and brackish waters, at environmentally relevant concentrations. Ash triggered the oxidative stress response of the bivalves and promoted metal accumulation. The expected increase in wildfire danger and burnt areas due to climate warming [28], allied to the likely concomitant exposure to other environmental contaminants and their interaction [72], raises concern about the effects of post-fire runoff to bivalve species, hence highlighting the need to develop future studies. Considering the high tolerance of *C. edule* to environmental contaminants and having in mind that the metal accumulation is site- and species-specific (e.g., [53]), further studies should be carried out with other aquatic species, using a multi-species approach. Both species used in the present study have a broad distribution and, like other bivalves, exhibit a high capacity to accumulate chemicals from their environment. In addition, the influence of biotic factors (such as age and physiological status) and abiotic factors (such as temperature and the concomitant presence of contaminants) should be taken into consideration as they affect the biochemical response [33] and metal accumulation by the organisms [24,73]. Both biotic and abiotic factors were controlled in the present study, which is not the case under a real exposure scenario. These factors might have a pronounced effect on the metal accumulation and, consequently, on the dietary risk for humans and other species feeding on these organisms. Additionally, future studies should consider other ash types, for example, from different types of vegetation or different fire severity, since these factors are known to influence the ash chemical composition, and consequently their toxicity to aquatic species [1]. Moreover, other endpoints, such as effects on the nutritional value of the ash-exposed organisms, should also be addressed to obtain a better understanding of wildfire ash on filter feeders.

## 4. Conclusions

The present study is the first to compare the sensitivity of freshwater and marine bivalves to wildfire ash. Bivalves are sensitive biomonitors for metal pollution due to their ability to uptake and accumulate these elements and are therefore ideal to assess the effects of exposure to wildfire ash. *C. fluminea* and *C. edule* differed in their response to AEAs exposure. Regarding the antioxidant defense system, *C. fluminea* was more sensitive than *C. edule*, showing a pronounced increase of tGPx activity with increasing AEA concentrations. 

Despite the metal body burden being considerable in field organisms of both species, exposure to AEAs led to accumulation of Cd in *C. fluminea* and Cu in *C. edule*. Still, only *C. edule* raised concerns for human consumption, as As concentrations were above the standard guidelines. Noteworthily, such a dietary risk was not associated with ash exposure. However, exposure to ash increased the dietary risk posed by Ni, considering organisms exposed to 100% AEA, given that the quantity of cockles that has to be consumed per week to exceed PTWI was below 1.0 kg. Moreover, metal concentrations in *C. edule* tended to be higher in organisms exposed to 100% AEA than control organisms. These results show that exposure to ash-associated contaminants at environmentally relevant concentrations can increase the metal body burden of cockles, with consequent risks for species feeding on them, namely for human health. We cannot discard the possibility that exposure during longer periods, or to higher ash concentrations, could lead to increased internal metal concentrations. Future studies should address these aspects and consider variables related to ash composition and species sensitivity, aiming to gain a comprehensive understanding of the real environmental threat of post-fire ash runoff to aquatic biota, both in the perspective of protection of coastal and inland water bodies and of assessing dietary risk regarding human consumption.

## Figures and Tables

**Table 1:** Average metal concentrations (µg L^−1^) in the test media (freshwater and brackish water, respectively for *C. fluminea* and *C. edule*), immediately after preparation (new medium) and after a 48 h exposure period (48 h old medium) at different aqueous extracts of ash (AEAs) concentrations (0, 12.5, 25, 50, and 100%). The quantification limit (QL) of the different elements for each medium is also presented. BQL—below quantification limit. Standard deviation is presented within brackets.

			V	Cr	Mn	Fe	Co	Ni	Cu	Zn	As	Cd	Pb
AEA in freshwater (%)	new medium	QL (µg L^−1^)	1	1	0.3	10	0.1	1	2	2	2	0.1	0.1
		0%	BQL	BQL	BQL	BQL	BQL	BQL	BQL	3.03 (0.59)	BQL	BQL	BQL
		12.5%	1.17 (0.058)	1.2 (0.10)	0.33	BQL	0.10	BQL	4.5	5.4 (4.67)	BQL	BQL	BQL
		25%	1.37 (0.058)	1.3 (0.00)	BQL	BQL	0.14 (0.006)	BQL	2.3	2.4	BQL	BQL	BQL
		50%	2.03 (0.55)	1.83 (0.38)	0.43	BQL	0.22 (0.042)	2.15 (1.34)	3.7 (0.62)	2.5	BQL	BQL	BQL
		100%	2.93 (0.67)	2.27 (0.64)	2.93 (2.51)	10.5	0.33 (0.046)	2.67 (1.85)	4.87 (0.76)	BQL	BQL	BQL	BQL
	48 h old medium	0%	BQL	BQL	BQL	BQL	0.17 (0.03)	BQL	BQL	13.7 (0.58)	BQL	BQL	BQL
		12.5%	BQL	BQL	BQL	BQL	0.18 (0.055)	BQL	BQL	9.6 (3.81)	BQL	BQL	BQL
		25%	BQL	BQL	BQL	BQL	0.18 (0.035)	BQL	BQL	5.93 (2.56)	BQL	BQL	BQL
		50%	1.3 (0.14)	2.00 (0.61)	BQL	BQL	0.31 (0.067)	BQL	2.73 (0.15)	39.5 (9.19)	BQL	BQL	BQL
		100%	2.55 (0.21)	2.87 (1.45)	0.33	BQL	0.49 (0.25)	BQL	7.23 (1.14)	79.5 (19.09)	BQL	BQL	BQL
AEA in brackish water (%)	new medium	QL (µg L^−1^)	40	20	4	150	2	50	40	40	50	2	2
		0%	BQL	BQL	BQL	BQL	BQL	BQL	BQL	BQL	BQL	BQL	BQL
		12.5%	BQL	BQL	6.0 (2.65)	BQL	BQL	BQL	BQL	BQL	BQL	BQL	BQL
		25%	BQL	BQL	7.0 (1.0)	BQL	BQL	BQL	BQL	BQL	BQL	BQL	BQL
		50%	BQL	BQL	5.5 (0.71)	BQL	2.0	BQL	BQL	BQL	BQL	BQL	BQL
		100%	BQL	BQL	12.7 (4.16)	BQL	2.0 (0.00)	BQL	BQL	BQL	BQL	BQL	BQL
	48 h old medium	0%	BQL	BQL	BQL	BQL	2.0	BQL	BQL	BQL	BQL	BQL	BQL
		12.5%	BQL	BQL	BQL	BQL	BQL	BQL	BQL	BQL	BQL	BQL	BQL
		25%	BQL	BQL	BQL	BQL	BQL	BQL	BQL	BQL	BQL	BQL	BQL
		50%	BQL	BQL	BQL	BQL	BQL	BQL	BQL	BQL	BQL	BQL	BQL
		100%	BQL	BQL	BQL	BQL	BQL	BQL	BQL	BQL	BQL	BQL	BQL

**Table 2:** Average metal concentrations (mg kg fresh weight^−1^) in *Corbicula fluminea* and *Cerastoderma edule* after 96 h of exposure to aqueous ash extracts (AEA) at different concentrations (0, 12.5, 25, 50, and 100%). Values for the bivalves immediately after arriving to the laboratory (reflecting the field condition) and at the beginning of the experiment are also presented, for comparison purposes. Standard deviation is presented within brackets. The maximum levels (ML) in shellfish (mg kg fresh weight^−1^) defined by the EFSA (European Food Safe Authorities [68]) and the FSANZ (Food Standards Australia and New Zealand [69]) are also presented. Treatments showing significant differences relative to the control (AEA 0%) are identified with an asterisk and highlighted in bold. Values exceeding the ML are highlighted in grey.

			V	Cr	Mn	Fe	Co	Ni	Cu	Zn	As	Cd	Pb
*Corbicula fluminea*	field				5.08 (1.41)	132.05 (18.88)	0.13 (0.02)		12.07 (1.35)	37.75 (14.74)		<0.03 ^a^	<0.03 ^a^
	t = 0 h				3.43 (2.32)	51.19 (11.69)	0.11 (0.03)		9.44 (1.78)	14.53 (1.37)		0.04 (0.01)	0.13 (0.16)
	t = 96 h	0%	0.67 (0.03)		2.31 (0.54)	49.46 (3.62)	0.08 (0.008)		7.28 (1.08)	14.4 (0.34)		<0.27 ^a^	<0.27 ^a^
		12.5%	0.75 (0.017)		2.26 (0.60)	42.66 (3.86)	0.07 (0.018)		5.22 (0.29) *	12.12 (1.14)		0.04 (0.009)	0.03 (0.001)
		25%			2.3 (0.56)	40.33 (2.78) *	0.1 (0.005)		5.48 (0.63) *	12.57 (1.57)		0.04 (0.004)	0.04 (0.008)
		50%			1.59 (0.35)	38.67 (6.52) *	0.1 (0.027)		5.27 (0.75) *	12.71 (0.98)		0.05 (0.02)	0.03 (0.004)
		100%			1.36 (0.23) *	33.98 (1.72) *	0.09 (0.020)		5.12 (0.86) *	10.81 (1.86) *		0.03 (0.005)	0.04 (0.025)
*Cerastoderma edule*		field	0.13 (0.02)	0.08 (0.02)	0.74 (0.17)	50.95 (19.71)	0.11 (0.02)	1.65 (0.07)	0.49 (0.09)	5.74 (0.51)	1.51 (0.17)	0.03 (0.02)	0.06 (0.03)
		t = 0 h		< 0.08 ^a^	0.61 (0.16)	35.08 (3.91)	0.14 (0.05)	1.93 (0.8)	0.56 (0.05)	7.36 (0.89)	1.76 (0.08)	0.03 (0.01)	0.04 (0.01)
	t = 96 h	0%		< 0.07 ^a^	0.46 (0.15)	35.54 (4.16)	0.16 (0.022)	2.00 (0.062)	0.53 (0.037)	8.12 (0.99)	1.71 (0.07)	0.04 (0.007)	0.03 (0.006)
		12.5%		< 0.07 ^a^	0.56 (0.11)	35.76 (6.17)	0.13 (0.022)	2.16 (0.43)	0.56 (0.053)	8.09 (0.99)	1.63 (0.04)	0.04 (0.015)	0.04 (0.012)
		25%		0.07 (0.01)	0.52 (0.10)	34.75 (4.23)	0.14 (0.026)	1.83 (0.46)	0.55 (0.042)	7.04 (0.89)	1.70 (0.12)	0.04 (0.013)	0.02 (0.005)
		50%		0.08 (0.01)	0.59 (0.10)	37.16 (4.75)	0.12 (0.035)	2.03 (0.44)	0.61 (0.072)	7.29 (0.23)	1.82 (0.088)	0.03 (0.007)	0.03 (0.005)
		100%		0.08 (0.01)	0.72 (0.15)	39.22 (9.38)	0.16 (0.073)	2.63 (0.80)	0.58 (0.061)	7.61 (0.94)	1.71 (0.098)	0.04 (0.01)	0.03 (0.005)
ML		EFSA	-	-	-	-	-	-	-	-	-	1.0	1.5
		FSANZ	-	-	-	-	-	-	-	-	1	2	2

^a^ metal concentration values were below the quantification limit. To allow statistical analyses, the presented values were determined considering the individual’s weight and assuming a metal concentration equal to the quantification limit.

## Data Availability

All data supporting the results of the present study are reported in the manuscript or as Supplementary Information.

## Supplementary Materials

The following supporting information can be downloaded at: https://www.mdpi.com/article/10.3390/ijerph20021326/s1, Table S1: Physicochemical properties of the media used in the ecotoxicological experiments (freshwater and brackish water, respectively for *Corbicula fluminea* and *Cerastoderma edule*), both immediately after preparation (new medium) and after a 48 h exposure period (48 hold medium), regarding exposure to aqueous extracts of ash (AEAs) at different concentrations (0, 12.5, 25, 50 and 100%). Standard deviation is presented within brackets; Table S2: Quantification limit (QL) of the different polycyclic aromatic hydrocarbons (PAHs) determined in both the freshwater and the brackish media; Table S3: Average metal concentrations (mg. g dry weight^−1^) in *Corbicula fluminea* and *Cerastoderma edule* after 96 h of exposure to aqueous extracts of ash (AEAs) at different concentrations (0, 12.5, 25, 50 and 100%). Values for the bivalves immediately after arriving to the laboratory (reflecting the field condition) are also presented, for comparison purposes. Standard deviation is presented within brackets. Treatments showing significant differences relative to the control (AAE 0%) are identified with an asterisk and highlighted in bold; Table S4: Summary of the concentration of metals accumulated by *Corbicula fluminea* and *Cerastoderma edule* (soft tissue), expressed as µg g^−1^ dry weight), in different sites; Table S5: Summary of the statistical analysis comparing the metal body burden (expressed in mg. kg dry weight^−1^) of both *Corbicula fluminea* and *Cerastoderma edule* after 96 h of exposure to aqueous extracts of ash (AEAs) at different concentrations (0, 12.5, 25, 50 and 100%). *p*-values denoting statistically significant differences are highlighted in bold. U represents the result of the Mann-Whitney test, whereas t represents the result of the Student’s *t*-test; Table S6: Amount (kg) of clams (*Corbicula fluminea*) and cockles (*Cerastoderma edule*) that has to be consumed per week to exceed PTWI (kg week^−1^, fresh weight). Values below 1 kg are highlighted in bold. Values for V, Cr, Mn, Fe and Co are not presented as no PTWI values are reported in JECFA (2004). Calculations were performed based on the total metal concentration; Figure S1: Water content of the soft tissues of *Corbicula fluminea* and *Cerastoderma edule* individuals after 96 h of exposure to aqueous extracts of ash (AEA) at different concentrations (0, 12.5, 25, 50 and 100%). Bars represent mean values and the error bars represent standard deviation (*n* = 5 per treatment); Figure S2: Exposure media used in the experiments with *Corbicula fluminea* both immediately after preparation (A) and during the experiment (B). Note the increased coloration and suspended particles with increasing concentration (0, 12.5, 25, 50 and 100%) of the aqueous extracts of ash (AEAs). References [74,75,76,77,78] are cited in the Supplementary Materials.
