# Supplementary material for: Do Freshwater and Marine Bivalves Differ in Their Response to Wildfire Ash? Effects on the Antioxidant Defense System and Metal Body Burden
Source: Int J Environ Res Public Health. 2023 Jan 11;20(2):1326. doi: 10.3390/ijerph20021326 (PMC9859076; doi:10.3390/ijerph20021326)

Do freshwater and marine bivalves differ in their response to wildfire ash? Effects on the antioxidant defense system and metal body burden

Fátima Jesus [a], Filipa Mesquita [b], Elisa Virumbrales Aldama [c], Ana Marques [b], Ana M. M. Gonçalves [b,d,*], Luísa Magalhães[b], António J. A. Nogueira [b], Ana Ré [a], Isabel Campos [a], Joana Luísa Pereira [b], Fernando J. M. Gonçalves [b], Nelson Abrantes [a], Dalila Serpa [a]

[a] CESAM - Centre for Environmental and Marine Studies, Department of Environment and Planning, University of Aveiro, 3810-193 Aveiro, Portugal

[b] CESAM - Centre for Environmental and Marine Studies, Department of Biology, University of Aveiro, 3810-193 Aveiro, Portugal

[c] Faculty of Veterinary and Experimental Sciences, Catholic University of Valencia, Calle Guillem de Castro 94, 46001 Valencia, Spain

[d] University of Coimbra, MARE-Marine and Environmental Sciences Centre / ARNET - Aquatic Research Network, Department of Life Sciences, 3000-456 Coimbra, Portugal

* Correspondence: amgoncalves@uc.pt; Tel.: +351-239-240-700 (ext. 262-286)

Supplementary Material

Index

Tables

Table S1: Physicochemical properties of the media used in the ecotoxicological experiments (freshwater and brackish water, respectively for *Corbicula fluminea* and *Cerastoderma edule*), both immediately after preparation (new medium) and after a 48 h exposure period (48 hold medium), regarding exposure to aqueous extracts of ash (AEAs) at different concentrations (0, 12.5, 25, 50 and 100%). Standard deviation is presented within brackets.

Table S2: Quantification limit (QL) of the different polycyclic aromatic hydrocarbons (PAHs) determined in both the freshwater and the brackish media.

Table S3: Average metal concentrations (mg. g dry weight^{-1}) in *Corbicula fluminea* and *Cerastoderma edule* after 96 h of exposure to aqueous extracts of ash (AEAs) at different concentrations (0, 12.5, 25, 50 and 100%). Values for the bivalves immediately after arriving to the laboratory (reflecting the field condition) are also presented, for comparison purposes. Standard deviation is presented within brackets. Treatments showing significant differences relative to the control (AAE 0%) are identified with an asterisk and highlighted in bold.

Table S4: Summary of the concentration of metals accumulated by *Corbicula fluminea* and *Cerastoderma edule* (soft tissue), expressed as μg g^{-1} dry weight), in different sites.

Table S5: Summary of the statistical analysis comparing the metal body burden (expressed in mg. kg dry weight^{-1}) of both *Corbicula fluminea* and *Cerastoderma edule* after 96 h of exposure to aqueous extracts of ash (AEAs) at different concentrations (0, 12.5, 25, 50 and 100%). P-values denoting statistically significant differences are highlighted in bold. U represents the result of the Mann-Whitney test, whereas t represents the result of the Student's t-test.

Table S6: Amount (kg) of clams (*Corbicula fluminea*) and cockles (*Cerastoderma edule*) that has to be consumed per week to exceed PTWI (kg. week^{-1}, fresh weight). Values below 1 kg are highlighted in bold. Values for V, Cr, Mn, Fe and Co are not presented as no PTWI values are reported in JECFA (2004). Calculations were performed based on the total metal concentration.

Figures

Figure S1: Water content of the soft tissues of *Corbicula fluminea* and *Cerastoderma edule* individuals after 96 h of exposure to aqueous extracts of ash (AEA) at different concentrations (0, 12.5, 25, 50 and 100%). Bars represent mean values and the error bars represent standard deviation (n = 5 per treatment).

Figure S2: Exposure media used in the experiments with *Corbicula fluminea* both immediately after preparation (A) and during the experiment (B). Note the increased coloration and suspended particles with increasing concentration (0, 12.5, 25, 50 and 100%) of the aqueous extracts of ash (AEAs).

Table S1: Physicochemical properties of the media used in the ecotoxicological experiments (freshwater and brackish water, respectively for *Corbicula fluminea* and *Cerastoderma edule*), both immediately after preparation (new medium) and after a 48 h exposure period (48 hold medium), regarding exposure to aqueous extracts of ash (AEAs) at different concentrations (0, 12.5, 25, 50 and 100%). Standard deviation is presented within brackets.

		AAE (%)	pH	dissolved oxygen (mg L^{-1})	Conductivity (µS cm^{-1})	Salinity (PSU)
AAE in freshwater (%)	new medium	0%	6.99 (0.24)	9.64 (0.04)	405 (154)	0.0 (0.0)
		12.50%	7.39 (0.18)	9.62 (0.07)	633 (161)	0.05 (0.07)
		25%	7.61 (0.13)	9.56 (0.12)	840 (107)	0.10 (0.0)
		50%	7.8 (0.08)	9.5 (0.18)	1308 (122)	0.20 (0.0)
		100%	8.06 (0)	9.47 (0.15)	2228 (235)	0.40 (0.0)
	48 h old medium	0%	7.8 (0.23)	9.69 (0.22)	349 (187)	0.0 (0.0)
		12.50%	7.78 (0.39)	9.72 (0.17)	533 (264)	0.08 (0.05)
		25%	7.89 (0.43)	9.73 (0.2)	672 (295)	0.10 (0.0)
		50%	8.09 (0.44)	9.68 (0.24)	1080 (470)	0.25 (0.05)
		100%	8.20 (0.5)	9.61 (0.26)	1814 (866)	0.46 (0.05)

		AAE (%)	pH	dissolved oxygen (mg L^{-1})	Conductivity (mS cm^{-1})	Salinity (PSU)
AAE in brackish water (%)	new medium	0%	7.14 (0.06)	6.7 (0.19)	136 (12)	20.0 (0.0)
		12.50%	7.3 (0.05)	6.93 (0.01)	127 (1)	20.0 (0.0)
		25%	7.38 (0.02)	6.7 (0.23)	137 (12)	20.0 (0.14)
		50%	7.51 (0.01)	6.86 (0.45)	133 (19)	20.1 (0.14)
		100%	7.86 (0.1)	6.83 (0.06)	135 (3)	20.25 (0.07)
	48 h old medium	0%	7.88 (0.63)	6.44 (0.09)	149 (17)	20.29 (0.16)
		12.50%	8.39 (0.11)	6.39 (0.18)	155 (10)	20.75 (0.84)
		25%	8.19 (0.13)	6.29 (0.3)	165 (20)	20.6 (0.17)
		50%	8.11 (0.29)	6.5 (0.71)	163 (13)	20.89 (0.16)
		100%	8.11 (0.26)	6.04 (0.29)	176 (21)	20.91 (0.25)

Table S2: Quantification limit (QL) of the different polycyclic aromatic hydrocarbons (PAHs) determined in both the freshwater and the brackish media.

PAH	QL (µg L^{-1}) (freshwater)	QL (µg L^{-1}) (brackish water)
Naphthalene	0.05	0.01
Acenaphthylene	0.05	0.01
Acenaphthene	0.05	0.01
Fluorene	0.05	0.01
Phenanthrene	0.05	0.01
Anthracene	0.01	0.01
Fluoranthene	0.01	0.01
Pyrene	0.01	0.01
Benz(a)anthracene	0.01	0.01
Chrysene	0.01	0.01
Benzo(b)fluoranthene	0.01	0.01
Benzo(k)fluoranthene	0.01	0.01
Benzo(a)pyrene	0.01	0.007
Indeno(1,2,3-cd)pyrene	0.01	0.01
Dibenz(a,h)anthracene	0.01	0.01
Benzo(g,h,i)perylene	0.01	0.01

Table S3: Average metal concentrations (mg. g dry weight^{-1}) in *Corbicula fluminea* and *Cerastoderma edule* after 96 h of exposure to aqueous extracts of ash (AEAs) at different concentrations (0, 12.5, 25, 50 and 100%). Values for the bivalves immediately after arriving to the laboratory (reflecting the field condition) are also presented, for comparison purposes. Standard deviation is presented within brackets. Treatments showing significant differences relative to the control (AAE 0%) are identified with an asterisk and highlighted in bold.

		AAE (%)	V	Cr	Mn	Fe	Co	Ni	Cu	Zn	As	Cd	Pb
Corbicula fluminea		field			25.57 (4.76)	677.83 (137.39)	0.66 (0.05)		61.36 (3.13)	194.3 (85.42)		0.26 (0.04)	0.51 (0.13)
	t = 96 h	0 %	7.19 (7.19)		24.43 (8.05)	513.4 (54.56)	0.8 (0.10)		74.99 (6.98)	149.64 (14.99)		< 0.27 (0.045)[a]	< 0.27 (0.045)[a]
		12.5 %	9.08 (1.41)		25.77 (6.31)	487.8 (45.57)	0.83 (0.14)		**59.76 (4.32)** *	138.49 (12.09)		0.42 (0.08)	0.34 (0.03)
		25 %			24.78 (7.00)	432.3 (43.82)	1.06 (0.11)		**58.47 (4.01)** *	134.15 (12.69)		**0.44 (0.02)** *	0.37 (0.11)
		50 %			19.76 (4.94)	474.0 (58.62)	1.21 (0.29)		64.69 (6.23)	157.64 (23.93)		**0.54 (0.21)** *	0.38 (0.08)
		100 %		3.68 (0.89)	16.8 (2.63)	424.3 (75.02)	1.08 (0.31)		**62.79 (5.28)** *	132.11 (8.22)		0.33 (0.03)	0.44 (0.18)
Cerastoderma edule		field	1.42 (0.25)	0.79 (0.2)	7.4 (2.51)	498.81 (193.92)	1.12 (0.22)	16.25 (1.94)	4.93 (1.47)	56.26 (6.28)	15.05 (3.76)	0.32 (0.13)	0.57 (0.24)
	t0= 96 h	0 %		< 0.57 [a]	3.61 (1.23)	277.6 (35.69)	1.24 (0.12)	15.62 (1.14)	4.16 (0.36)	63.49 (8.78)	13.33 (0.41)	0.28 (0.042)	0.26 (0.043)
		12.5 %		0.61 (0.07)	4.8 (0.88)	307.1 (46.43)	1.11 (0.20)	18.59 (3.45)	4.79 (0.40)	69.49 (7.31)	13.99 (0.33)	0.32 (0.12)	0.34 (0.093)
		25 %		0.62 (0.1)	4.62 (0.84)	309.9 (44.85)	1.26 (0.21)	16.22 (3.76)	4.88 (0.35)	62.55 (7.03)	15.1 (1.359)	0.34 (0.11)	0.21 (0.048)
		50 %		0.65 (0.09)	5.1 (1.38)	317.6 (39.79)	1.04 (0.39)	17.56 (4.83)	**5.21 (0.56)** *	62.37 (4.48)	15.61 (1.75)	0.27 (0.053)	0.24 (0.047)
		100 %		0.66 (0.12)	6.29 (1.29)	343.5 (92.5)	1.41 (0.69)	23 (7.44)	**5.07 (0.49)** *	66.53 (9.73)	14.9 (0.66)	0.34 (0.091)	0.25 (0.051)

[a)] metal concentration values were below the quantification limit. To allow statistical analyses, the presented values were determined considering the individuals weight and assuming a metal concentration equal to the quantification limit.

Table S4: Summary of the metal body burden in *Corbicula fluminea* and *Cerastoderma edule* (soft tissue), expressed as µg g^{-1} dry weight, in different sites.

	V	Cr	Mn	Fe	Co	Ni	Cu	Zn	As	Cd	Pb	site	condition	ref.
C. fluminea	-	-	25.57	677.83	0.66	-	61.36	194.3	-	0.26	0.51	Mira, Portugal	Field, reference site[a]	Present study
	0.42	1.12	19.90	-	2.28	2.90	126.10	150.70	6.79	0.79	0.83	Mira, Portugal	After depuration - Control conditions [a]	(Silva et al. 2016)
	-	2.67	-	-	-	-	51.2	142.4	3.49	3.06	0.6	Altamaha River, Georgia, USA	Field, different sites [a]	(Shoults-Wilson et al. 2009)
	-	2.89	10.2	322	0.46	2.81	17.2	57.4	1.57	0.14	0.66	Odiel River basin, Spain	Field, reference site	(Bonnail et al. 2019)
	-	12.86	230.17	4552.11	3.28	46.90	91.10	279.32	8.29	3.59	5.88	Liuyang River, China	Field, different sites [a]	(Jia et al. 2018)
	-	1.0-1.8	-	-	-	5.8-11	34-71	136-161	-	1.1-2.5	0.45-1.3	Minho estuary, Portugal	Field, different sites [a]	(Reis et al. 2014)
C. edule	1.42	0.79	7.4	498.8	1.12	16.2	4.93	56.3	15.0	0.32	0.57	Mira, Portugal	Field, reference site[a]	Present study
	-	1.08	11.15	246.71	0.83	3.06	3.54	27.50	2.85	0.06		Messina, Italy	Ganzirri Lake[a]	(Di Bella et al. 2013)

6.6	3.2	-	-	1.7	47	-	-	20	0.73	4.7	Tagus Estuary	Reference site [a]	(Marques et al. 2016)
7.5	2.0	-	-	2.0	41	-	-	33	0.67	9.9	Tagus Estuary	Polluted site [a]	(Marques et al. 2016)
-	-	-	-	-	-	8.77-22.6	58.3-115	-	1.57-2.37	16.5-18.9	Morocco (Mediterranean coast)	Field, different sites [a]	(Cheggour et al. 2001)
-	-	-	-	-	-	12.4	72.7	-	344	-	Gironde estuary, France	Bleu Médoc" fish farm [a]	(Baudrimont et al. 2005)
	-	-	-	-	84-117	12-34	68-160	17-23	1-2.8	3-8.2	Sado estuary, Portugal	Laboratorial exposure to sediments from different sites [a]	(Lobo et al. 2010)
	≈ 0.2-7.9	-	-	-	≈ 15-38	≈ 3.6-10.2	≈ 50-100	-	≈ 0.35-1.6	≈ 0.2-3.6	Souss Estuary, Morocco	Field	(Anajjar et al. 2008)

[a)] mean values

References

Anajjar, E. M., J.-F. Chiffoleau, H. Bergayou, A. Moukrim, T. Burgeot, and M. Cheggour. 2008. Monitoring of Trace Metal Contamination in the Souss Estuary (South Morocco) Using the Clams Cerastoderma edule and Scrobicularia plana. Bulletin of Environmental Contamination and Toxicology **80**:283-288.

Baudrimont, M., J. Schäfer, V. Marie, R. Maury-Brachet, C. Bossy, A. Boudou, and G. Blanc. 2005. Geochemical survey and metal bioaccumulation of three bivalve species (Crassostrea gigas, Cerastoderma edule and Ruditapes philippinarum) in the Nord Médoc salt marshes (Gironde estuary, France). Science of the Total Environment **337**:265-280.

Bonnail, E., F. Macías, and V. Osta. 2019. Ecological improvement assessment of a passive remediation technology for acid mine drainage: Water quality biomonitoring using bivalves. Chemosphere **219**:695-703.

Cheggour, M., A. Chafik, W. J. Langston, G. R. Burt, S. Benbrahim, and H. Texier. 2001. Metals in sediments and the edible cockle Cerastoderma edule from two Moroccan Atlantic lagoons: Moulay Bou Selham and Sidi Moussa. Environmental Pollution **115**:149-160.

Di Bella, G., V. L. Turco, A. G. Potortì, R. Rando, P. Licata, and G. Dugo. 2013. Statistical analysis of heavy metals in Cerastoderma edule glaucum and Venerupis aurea laeta from Ganzirri Lake, Messina (Italy). Environmental Monitoring and Assessment **185**:7517-7525.

Jia, Y., L. Wang, Z. Qu, and Z. Yang. 2018. Distribution, contamination and accumulation of heavy metals in water, sediments, and freshwater shellfish from Liuyang River, Southern China. Environmental Science and Pollution Research **25**:7012-7020.

Lobo, J., P. M. Costa, S. Caeiro, M. Martins, A. M. Ferreira, M. Caetano, R. Cesário, C. Vale, and M. H. Costa. 2010. Evaluation of the potential of the common cockle (Cerastoderma edule L.) for the ecological risk assessment of estuarine sediments: bioaccumulation and biomarkers. Ecotoxicology **19**:1496-1512.

Marques, A., D. Piló, O. Araújo, F. Pereira, S. Guilherme, S. Carvalho, M. A. Santos, M. Pacheco, and P. Pereira. 2016. Propensity to metal accumulation and oxidative stress responses of two benthic species (Cerastoderma edule and Nephtys hombergii): are tolerance processes limiting their responsiveness? Ecotoxicology **25**:664-676.

Reis, P. A., L. Guilhermino, C. Antunes, and R. G. Sousa. 2014. Assessment of the ecological quality of the Minho estuary (Northwest Iberian Peninsula) based on metal concentrations in sediments and in Corbicula fluminea. Limnetica.

Shoults-Wilson, W. A., J. T. Peterson, J. M. Unrine, J. Rickard, and M. C. Black. 2009. The Asian clam Corbicula fluminea as a biomonitor of trace element contamination: accounting for different sources of variation using an hierarchical linear model. Environmental Toxicology Chemistry: An International Journal **28**:2224-2232.

Silva, V., N. Abrantes, R. Costa, J. J. Keizer, F. Gonçalves, and J. L. Pereira. 2016. Effects of ash-loaded post-fire runoff on the freshwater clam Corbicula fluminea. Ecological Engineering **90**:180-189.

Table S5: Summary of the statistical analysis comparing the metal body burden (expressed in mg. kg dry weight^{-1}) of both *Corbicula fluminea* and *Cerastoderma edule* after 96 h of exposure to aqueous extracts of ash (AEAs) at different concentrations (0, 12.5, 25, 50 and 100%). P-values denoting statistically significant differences are highlighted in bold. U represents the result of the Mann-Whitney test, whereas t represents the result of the Student's t-test.

	Test	p
Body burden - Mn	$U = 0.000$<br>$n = 20; 20$	**$p \leq 0.001$**
Body burden - Fe	$t = 8.505$<br>38 degrees of freedom	**$p < 2.6x10^{9}$**
Body burden - Co	$U = 115.000$<br>$n = 20; 20$	**$p = 0.022$**
Body burden - Cu	$U = 0.000$<br>$n = 20; 20$	**$p \leq 0.001$**
Body burden - Zn	$U = 0.000$<br>$n = 20; 20$	**$p \leq 0.001$**
Body burden - Cd	$U = 52.000$<br>$n = 15; 20$	**$p = 0.001$**
Body burden - Pb	$U = 15.000$<br>$n = 12; 19$	**$p \leq 0.001$**

Table S6: Amount (kg) of clams (*Corbicula fluminea*) and cockles (*Cerastoderma edule*) that has to be consumed per week to exceed PTWI (kg. week^{-1}, fresh weight). Values below 1 kg are highlighted in bold. Values for V, Cr, Mn, Fe and Co are not presented as no PTWI values are reported in JECFA (2004). Calculations were performed based on the total metal concentration.

	AAE (%)	Ni	Cu	Zn	As	Cd	Pb
PTWI [a)]		0.035	3.5	7	0.015	0.007	0.025
Corbicula fluminea	field	-	20.29	12.98	-	14.64	52.29
	0%	-	33.67	34.03	-		
	12.5%	-	46.9	40.43	-	13.28	58.3
	25%	-	44.69	38.98	-	11.76	50.41
	50%	-	46.53	38.56	-	10.86	57.07
	100%	-	47.84	45.32	-	18.16	46.49
Cerastoderma edule	field	1.48	495.06	85.37	**0.69**	14.79	29.85
	0%	1.23	459.61	60.32	**0.61**	13.82	52.03
	12.5%	1.13	440.32	60.6	**0.65**	13.03	43.98
	25%	1.34	446.2	69.61	**0.62**	12.81	73.96
	50%	1.21	401.61	67.22	**0.58**	15.33	63.5
	100%	**0.93**	420.79	64.39	**0.61**	12.72	60.17

[a)] Provisional Tolerable Weekly Intake, considering a 70 kg adult; expressed as mg. kg^{-1}. week^{-1}, based on JECFA values (JECFA, 2004)

Reference

JECFA (Food, Agriculture Organization and World Health Organization, FAO/WHO). Safety evaluation of certain food additives and contaminants. 61st meeting of the joint FAO/WHO expert committee on food additives. WHO Food Additives Series. Geneva: International Programme on Chemical Safety, World Health Organization; 2004.

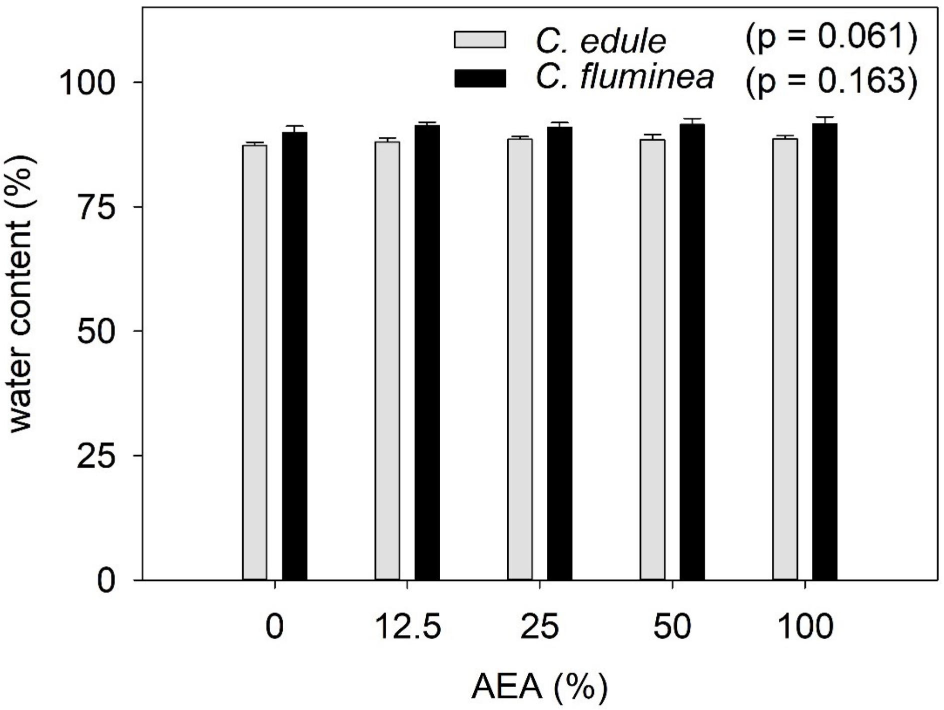

Figure S1: Water content [a)] of the soft tissues of *Corbicula fluminea* and *Cerastoderma edule* individuals after 96 h of exposure to aqueous extracts of ash (AEA) at different concentrations (0, 12.5, 25, 50 and 100%). Bars represent mean values and the error bars represent standard deviation (n = 5 per treatment).

[a)] determined following the expression:

$$water\ content = \frac{soft\ tissue\ wet\ weight - soft\ tissue\ dry\ weight}{soft\ tissue\ wet\ weight} \times 100$$

Figure S2: Exposure media used in the experiments with *Corbicula fluminea* both immediately after preparation (A) and during the experiment (B). Note the increased coloration and suspended particles with increasing concentration (0, 12.5, 25, 50 and 100%) of the aqueous extracts of ash (AEAs).

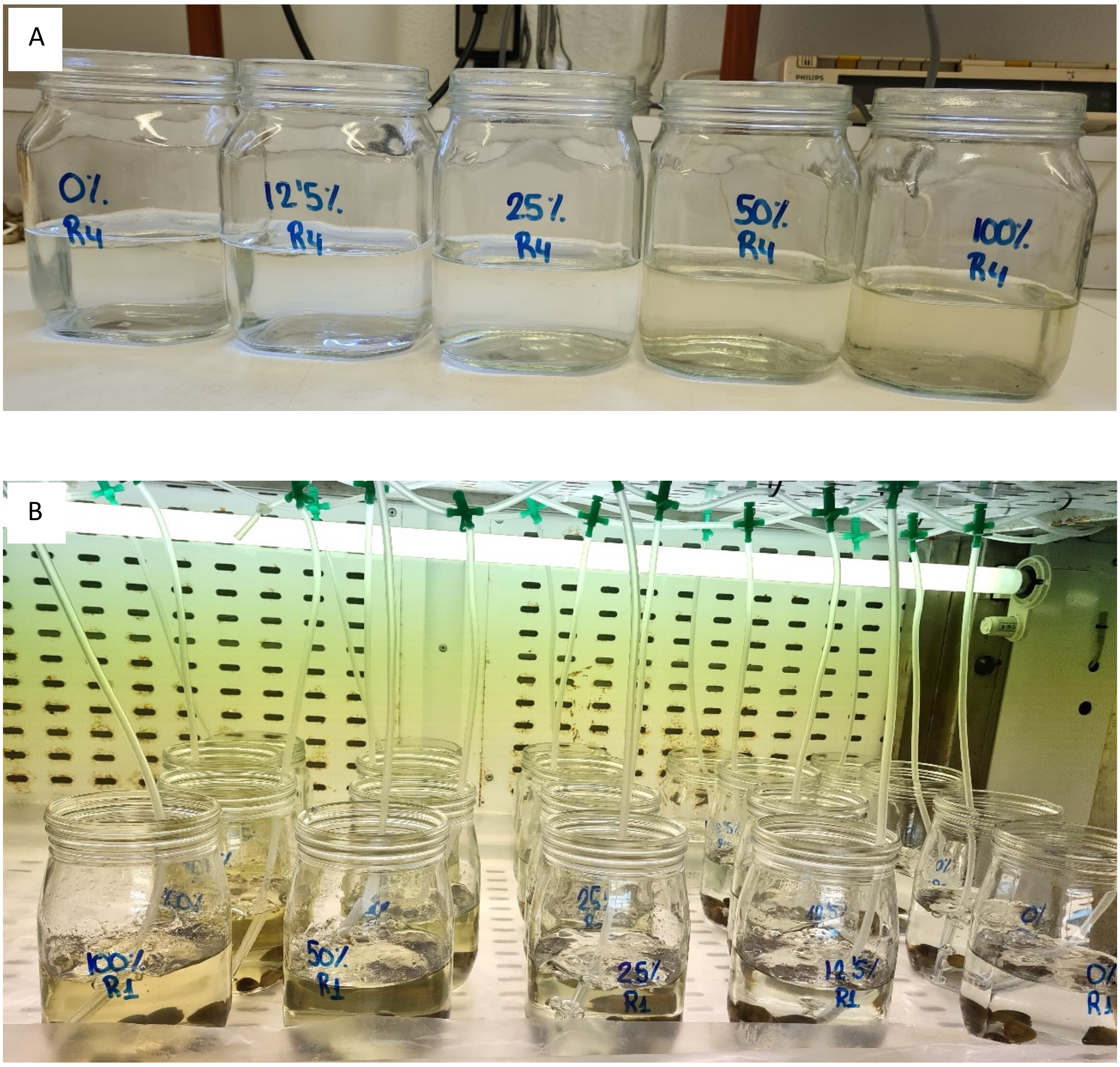

Supplement: Supplementary file 1 [file ijerph-20-01326-s001.zip › ijerph-2150280-supplementary.pdf]
